# The Core Dimensions of Integrated Care: A Literature Review to Support the Development of a Comprehensive Framework for Implementing Integrated Care

**DOI:** 10.5334/ijic.4198

**Published:** 2018-08-08

**Authors:** Laura G. González-Ortiz, Stefano Calciolari, Nick Goodwin, Viktoria Stein

**Affiliations:** 1 Università della Svizzera Italiana, IdEP Institute, CH; 2 International Foundation for Integrated Care, UK

**Keywords:** benchmarking, chronic conditions, framework, implementation science, integrated care, literature review

## Abstract

**Objective:**

As part of the EU-funded Project INTEGRATE, the research sought to develop an evidence-based understanding of the key dimensions and items of integrated care associated with successful implementation across varying country contexts and relevant to different chronic and/or long-term conditions. This paper identifies the core dimensions of integrated care based on a review of previous literature on the topic.

**Methodology:**

The research reviewed literature evidence from the peer-reviewed and grey literature. It focused on reviewing research articles that had specifically developed frameworks on integrated care and/or set out key elements for successful implementation. The search initially focused on three main scientific journals and was limited to the period from 2006 to 2016. Then, the research snowballed the references from the selected published studies and engaged leading experts in the field to supplement the identification of relevant literature. Two investigators independently reviewed the selected articles using a standard data collection tool to gather the key elements analyzed in each article.

**Results:**

A total of 710 articles were screened by title and abstract. Finally, 18 scientific contributions were selected, including studies from grey literature and experts’ suggestions. The analysis identified 175 items grouped in 12 categories.

**Conclusions:**

Most of the key factors reported in the literature derive from studies that developed their frameworks in specific contexts and/or for specific types of conditions. The identification and classification of the elements from this literature review provide a basis to develop a comprehensive framework enabling standardized descriptions and benchmarking of integrated care initiatives carried out in different contexts.

## Introduction

This paper presents findings from the “International Check” work package of Project INTEGRATE that was funded within the EU 7^th^ Framework Program (EU Grant Agreement 305821; see http://projectintegrate.eu.com). The overall purpose of the work package was to develop an evidence-based framework on the key dimensions and items of integrated care associated with successful implementation. Moreover, the purpose of such a framework was designed to support decision-makers in the effective design and implementation of integrated care programs[1].

Several studies have contributed to the development of theoretical frameworks for integrated care implementation (e.g. [2,3,4,5,6,7,8,9,10]). The articles and technical reports published on the topic identify factors or structures of elements fostering care integration, most often for people suffering from chronic and/or long-term conditions. In addition, most of these studies focus on a specific context of implementation (e.g. in coordinating services around people with a chronic illness) and so do not appreciate the influential role that contextual factors in care integration can play in determining outcomes (e.g. of finances, cultures, organizational forms etc.) [11]. In the former case, the resulting framework or list of key factors is likely to be tailored for the selected setting. In the latter case, it is hard to disentangle the context dependence of the analytical proposal.

Context is very important in evaluating the implementation of complex service innovations like integrated care and so any framework must be robust enough to understand the intricate interplay between multi-component interventions across contexts and settings [11,12,13]. The COMIC Model for the comprehensive evaluation of integrated care interventions [11], for example, has illustrated how the use or realistic synthesis to study the interplay between contexts, mechanisms and outcomes can bring insights into understanding how and why integrated care interventions succeed or fail.

Hence, to understand the complex and dynamic issues at play in the implementation of integrated care a more comprehensive framework is necessary that helps to benchmark initiatives across different contexts and condition-specific population groups. The task is a challenging one and cannot disregard the accumulated knowledge in the field. Therefore, as a first step in this direction, it is paramount to analyze and summarize the findings from previous studies in this respect. This can provide the basis for a more comprehensive approach to understanding how integrated care may be implemented by building on the evidence available in the extant literature of integrated care.

This paper presents a comprehensive, non-systematic, review of the extant literature on care integration design and implementation. Coherently with the objectives of Project INTEGRATE, this study purposefully focuses on initiatives targeting patients affected by chronic diseases (COPD and diabetes) and people living with geriatric and/or mental health conditions. The purpose of this paper, therefore, has been to build a more comprehensive understanding of the factors and elements associated with successful integrated care implementation as a precursor to the development of a new conceptual framework. The objectives of this paper are: (1) to identify the most important factors influencing the success of an initiative of care integration across different contexts; and (2) to classify and summarize the identified evidence according to the extant literature in the field.

## Background

To ensure a common understanding of the concept, Project INTEGRATE utilised Kodner and Spreeuwenberg’s definition of integrated care as: ‘a coherent set of methods and models on the funding, administrative, organisational, service delivery and clinical levels designed to create connectivity, alignment and collaboration within and between the cure and care sectors’ [14]. Hence, the main purpose of integrated care interventions consists of reducing fragmentations in service delivery and to foster both comprehensiveness of care and better care co-ordination around people’s needs. Many frameworks have been developed over time to understand the key elements, or building blocks, of integrated care [15].

One of the most well-known is the Chronic Care Model (CCM) that was developed from a Cochrane systematic review [10]. This work developed a comprehensive framework for the organization of healthcare to improve outcomes for people with chronic conditions. It identified six interrelated domains that should be considered to facilitate high-quality chronic disease care, thus improving health outcomes [3]. The CCM identifies the main areas of intervention to accomplish such a goal and enhance the health outcomes for specific target patients. In particular, its approach focuses on fostering an effective use of community resources; enabling patient self-management, nurturing evidence-based care and patient preferences, and leveraging on the use of supportive information technology [4].

Since the CCM focuses on the delivery of clinically oriented systems to patient it did not include many key aspects of care integration – for example, regarding health promotion and prevention, or indeed of rehabilitation and re-ablement. In this respect, Barr and colleagues proposed an evolution of the model, called the Expanded Chronic Model (ECCM) [2]. The ECCM includes elements specifically aimed to promote population health and encourage prevention by involving the community. Another variation is the Innovative Care for Chronic Conditions model (ICCC) [16]. Developed by the WHO as part of a ‘road map’ for health systems to deal with the rising burden of chronic illness, the ICCC placed a specific premium on prevention through ‘productive partnerships’ between patients and families, community partners and health care teams to create informed, prepared and motivated communities. In this respect, recent developments of integrated care initiatives, such as the patient-centred medical home (PCMH), have stressed the importance of delivering continuous, comprehensive and coordinated care in the context of people’s family and community [17].

The frameworks described above have primarily evolved from the USA and been confined in their thinking to *within* health systems. They have also not sought to identify key actions that decision-makers would need to implement integrated care effectively, such as governance and accountability, financing and incentives, or issues related to culture and values. However, other work has sought to address this. For example, a knowledge synthesis from Canada developed an influential paper entitled ‘ten principles of successful integrated systems’ [18].

More recently, Minkman et al. [7] carried out a study, based on a Delphi method, to identify and validate analytical key factors of care integration. The authors started by identifying elements from the literature. Then, they conducted a three-round Delphi study among a group of thirty-one experts, who provided comments to rank 175 elements in priority order. Then, the expert panel clustered the elements and discussed their content following a concept mapping procedure. Finally, the authors identified 89 relevant elements grouped into nine clusters. The results aim to develop a comprehensive quality management model for integrated care.

In another recent paper, Valentijn et al. [9] proposed a taxonomy to facilitate the description and comparison of different integrated care interventions. The taxonomy consists of 59 key elements resulting from a two-round Delphi study. This contribution is the development of a companion paper [8] in which the authors proposed a general model, known as the Rainbow Model of Integrated Care. Such model proposes six dimensions (in which the aforementioned 59 elements are grouped) and has a primary care perspective.

Compared to the CCM and the ECCM, the last two models are much more analytical in terms of the wider range of factors necessary to support the effective development of integrated care systems. Despite the differences they currently compete to propose an evidence-based perspective on the topic. Each has been developed for a different purpose and each varies in scope, for example, from the process of coordinating services around people with chronic conditions to enabling health and social care systems to operate more cohesively. For professionals and decision-makers tasked with designing and implementing integrated care for different client groups in a range of contexts and settings this potentially provides for confusion on the most appropriate frameworks and models they might use. This indicates that a more comprehensive framework – one that leverages the strengths of previously published studies but which enables an understanding of the core dimensions of integrated care across contexts and settings – is necessary to support decision-makers in the design and implementation of their integrated care programs across settings and differing client groups.

## Methodology

The authors searched for scientific studies published in peer-reviewed journals and the most important contributions in the grey literature (research reports and conference presentations). The adopted search strategy aimed to be efficient and flexible enough to include also important seminal contributions on the topic. Therefore, the authors agreed on starting with three specialized journals where peer-reviewed articles presenting frameworks of integrated care would most likely be cited, namely: the International Journal of Integrated Care (IJIC), the Journal of Integrated Care (JIC), and the International Journal of Coordinated Care^1^ (IJCC). The initial search was limited to the period 2006–2015.

The two authors from USI retrieved all the abstracts published in the selected period. Then, each one read half of the abstracts of all the articles to decide which contributions should be further analyzed. At this stage, the preliminary inclusion criteria consisted of two simple questions: (a) Does the contribution propose or analyze any framework aimed to explain the success or describe the implementation of integrated care initiatives? (b) Does the article propose or analyze any important aspect/s explaining the success of integrated care initiatives?

Each of the two researchers crosschecked the list of abstracts selected by her/his colleague until they reached an agreement. Afterwards, the two researchers read separately the full text of the selected articles to confirm their inclusion. They looked for contributions defining and operationalizing relevant elements of care integration or discussing the relationships between elements of care integration (inclusion criteria). They also applied the following exclusion criteria: (1) Limited to motivating the importance and goals of integrated care; (2) Limited to describing care integration properties and/or principles; (3) Limited to problems/gaps pf a specific case or national context; (4) Limited to a specific means/technology of integration (e.g. integrated care pathways); (5) Not normative (hard to use for assessing an initiative); (6) Limited to assessment of results of integrated care (not elements/strategies of care integration); (7) Review not based on a specific framework; (8) Not focused on chronic diseases or long-term conditions; (9) Non enough details (to allow for operationalization); (10) Based on another framework/model (and with no significant additional contribution); (11) Too much focused on a specific target population (e.g., elderly, minors, diabetic patients). Then, the two researchers compared the results of their selective analysis and found agreement when it was necessary to reconcile differences.

After this step, the two researchers analyzed the references of the included contributions looking for further relevant articles (snowballing). This approach was designed to identify previously published frameworks (including in the grey literature). The complete list of the identified contributions was sent to the other two authors from IFIC, who confirmed each item and suggested relevant contributions not included. All the researchers agreed on the new list. Then they performed hand searches looking for further articles/documents in selected databases (ScienceDirect, PubMed, and Medline) and collected suggestions from experts in the field. This range of experts included those within the research consortium plus the 13 members of the Advisory Board of Project INTEGRATE comprising leading academics, policy-makers, professional and commissioners across health, social care and public health disciplines (see: www.projectintegrate.eu.com/integrated-care-purpose).

Once all the authors agreed on the final list (with the earlies selected article published in 2002), the two researchers from USI carefully analyzed each selected contribution to identify the elements of the proposed framework or relevant aspects reported as factors fostering care integration. In some cases, the framework and/or the elements were quite evident, since they were listed or presented in a schematic way. For instance, Lyngsø et al. [6] categorized and listed the key elements in a table. In other contributions, the elements had to be meticulously identified into the text and extracted by the researchers. In such cases, both researchers independently identified and coded the elements from each selected document. Then, they cross-checked the results and found agreement on eventual differences.

The identified elements (hereafter referred as “items”) were gathered in a comprehensive list. Each item was written down textually to avoid misunderstandings in the next phase (validation). Supported by existing dimensions of the CCM, we drafted a table and placed each identified item into a corresponding category. Each item was associated with a consecutive number and the article/document where it was identified. Elements not fitting in any of the CCM dimensions were listed at the bottom of the table to be afterwards grouped into additional categories proposed by the researchers. It is important to mention that, in this study, we went for inclusiveness. Therefore, items quite similar but with non-identical wording were reported as a different item.

## Results

The database search, spanning over a decade in the publication history of the three selected journals, retrieved 710 peer-reviewed articles. The majority of these studies were subsequently excluded (679 articles) based on their title or abstract, because they did not meet the inclusion criteria. Therefore, we focused on the remaining 31 articles eligible for inclusion. After full text reading, based on the aforementioned exclusion criteria, 14 studies remained.

We found articles written by the same author(s) and discussing elements to foster integrated care already proposed in preceding companion(s) paper(s). In this case, we included only the most recent article, which generally confirms or further develops ideas/notions proposed in the previous one/s. We did not include any study from the references search (snowballing), because none of the identified articles did actually meet the predefined eligibility criteria.

**Figure 1:**
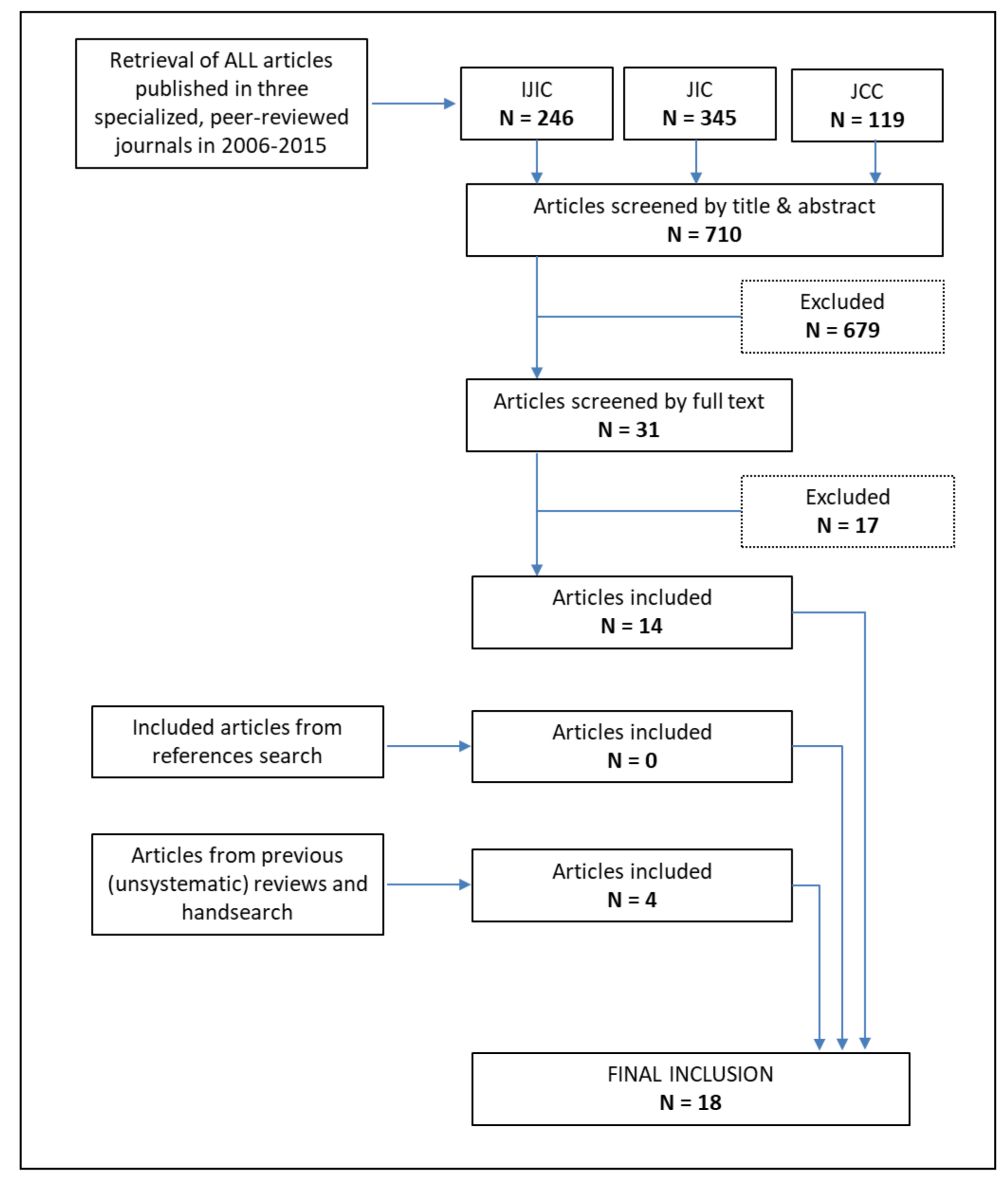
Flowchart of the literature selection process.

With regard to the grey literature and expert suggestions, we selected four studies. This resulted in 18 studies ultimately included in our review [4,5,6,8,11,13,14,15,16,17,18,19,20,21,22,23,24,25]. From each of the selected contributions, we identified and extracted items considered influential for care integration. We obtained a comprehensive list consisting of 175 items categorized in 12 domains. The first six domains are those proposed by the CCM: health care system, community resources and policies, self-management support, delivery system design, decision support, and clinical information system. In addition, we defined six further categories (leadership, governance, performance monitoring, organizational culture, contextual factors, and social capital) to group those elements that did not fit with any of the CCM domains. Figure 1 summarizes the selection process followed in the search and Table 1 the results of the search across the 12 domains.

**Table 1:** Results of the search across the identified 12 conceptual domains.

Domain	No.	Element/item	Reference

**Healthcare system**	1	Universal coverage or enrolled population with care free at point of use	[5,19]
	2	Emphasis on chronic and long-term care	[5,20]
	3	Emphasis on population health management	[5,21,22]
	4	Alignment of regulatory frameworks with goals of integrated care	[5,6,16]
	5	Data on chronic illnesses (eg. registries)	[23]
	6	Understand needs and priorities of local populations	[9,21,22,24]
	7	Mobilize and coordinate resources	[16,22]
	8	Adequate financing system linked with quality improvement	[22,23,25]
	9	Funding payment flexibilities to promote integrated care	[5]
	10	Allocating financial budgets for the implementation and maintenance of integrated care	[7,25]
	11	Funding of a program or service	[26]
	12	Changes to funding arrangements	[26]
	13	Finances for implementation and maintenance	[27]
	14	Reaching agreements on the financial budget for integrated care	[7]
	15	Prepaid capitation at various levels	[14]
	16	Financing mechanism allowing for pooling of funds across services	[6,14,19]
	17	Creating financial and regulatory incentives that encourage cooperation among health care providers	[6]

**Community resources and policies**	18	Integrate policies: collaboration/coordination across health-related policy fields (eg. environment, education, transportation, housing)	[9,16,25]
	19	Location policy	[9]
	20	Inter-organisational strategy	[6,9,20]
	21	Creating interdependence between organisations	[7,9]
	22	Reaching agreements on introducing and integrating new partners in the care chain	[7]
	23	Formal connections between organisations: varying from linkage with community to merging of organisations	[6,7,20,21,25,26]
	24	Achieving adjustments among care partners	[7]
	25	Reaching agreements about letting go care partner domains	[7]
	26	Reaching agreements among care partners on the consultation of experts and professionals	[7]
	27	Reaching agreements among care partners on managing client preferences	[7]
	28	Reaching agreements among care partners on scheduling client examinations and treatment	[7]
	29	Reaching agreements among care partners on discharge planning	[7]
	30	Making transparent the effects of the collaboration on the production of the care partners	[7]
	31	Structural meetings with external parties such as insurers, local governments and inspectorates	[7]
	32	Structural meetings of leaders of care-chain organizations	[7,21]
	33	Role of volunteers and third sector to support needs of patients and carers	[5,22]
	34	Building systems of care at the neighborhood level	[5,22]
	35	Building community awareness and trust with services (gives legitimacy to new approaches to care, and increase likelihood of appropriate, and earlier, referrals)	[5]
	36	Family caregivers (involvement and support)	[5,14,18,20,28]
	37	Coordinated home and community health	[5,22]
	38	Build resilience among carers to promote home-based care	[5,22]
	39	Raise awareness and reduce stigma	[16]
	40	Social value creation	[9]
	41	Provide complementary services	[16]

**Self-management support**	42	Patient education	[5,22]
	43	Patient empowerment	[23]
	44	Using self-management support methods as a part of integrated care	[5,7,9,21]
	45	Patient engagement and participation, i.e. patients provide input on various levels	[6,9,20,23]
	46	Electronic tools for patients to be engaged and active in self-management	[20,23]
	47	Patient navigation/clinical pathways	[20]
	48	Reminders for patients	[23,26]

**Delivery system design**	49	Paradigm shift from acute to chronic care and from reactive to proactive care delivery	[20,25]
	50	Population-based needs assessment: focus on defined population	[6,21,22,25]
	51	Defining the targeted client group	[5,7]
	52	Developing care programmes for relevant client subgroups	[7]
	53	Designing care for clients with multi- or co-morbidities	[7]
	54	Understand best ways to organize and implement care	[24]
	55	Collaborative involvement in planning, policy development and patient care delivery	[6]
	56	Service characteristics	[9]
	57	Co-location of services	[5,9,14,21,26]
	58	Specialized clinic or centres	[27]
	59	Patient-centered philosophy (focus on patients’ need)	[6,9,29]
	60	Promotion of functional independence and wellbeing, not just the management or treatment of medical symptoms (holistic focus)	[5,9,28]
	61	Commitment to the view that the patient is the customer	[6]
	62	Interaction between professional and client	[9]
	63	Care plans including collaborative goal setting between patients and clinicians	[9,20,26]
	64	Centralized information, referral and intake	[5,14]

**Delivery system design**	65	Single point of entry and a single point of contact for patients and carers	[5,7,21]
	66	Case management (relational continuity with a named coordinator)	[5,6,7,9,14,20,21,26,27]
	67	Case management	[5]
	68	Arrangements for priority access to another service	[26]
	69	Disease management	[14]
	70	Professional attitude and fulfilment of work as drivers of integration	[14]
	71	Multidisciplinary teamwork	[5,14,16,20,21,26,27,29]
	72	Developing a multi-disciplinary care pathway	[6,7,27]
	73	Creating interdependence between professionals (inter-professional networks)	[5,9,14,18,20]
	74	Teamwork (joint working) and care coordination	[5]
	75	Arrangements for facilitating communication	[7,26]
	76	Information sharing, planned/organised meetings	[20]
	77	Using a uniform language in the care chain	[7]
	78	Using uniform client-identification numbers within the care chain	[7]
	79	Shared assessment	[21,26]
	80	Coordinated or joint consultations	[26]
	81	Using feedback and reminders by professionals for improving care	[7,23,27]
	82	Agreements on referrals, discharge and transfer of clients through the care chain	[5,6,7,14]

**Delivery system design**	83	Clinical follow-up	[27]
	84	Continuity of care	[9,18,22,29]
	85	Assisted living/care support at home	[5,21]
	86	Service management (e.g., collective telephone numbers, counter assistance and 24-hour access)	[9,14,21]
	87	Medication management	[5]
	88	Essential and new pharmaceuticals and medical devices	[23]
	89	Collaboratively assessing bottlenecks and gaps in care	[7]
	90	An adequate workforce (in terms of number, competencies and distribution)	[5,9,22,23]
	91	Workforce educated and skilled in chronic care (graduate)	[5,6,16,20]
	92	Cross-training of staff (to ensure staff culture, attitudes, skills are complementary)	[6,7,9,14,16,22]
	93	Reaching agreements among care partners on tasks, responsibilities and authorizations	[7,23]
	94	Establishing the roles and tasks of multidisciplinary team members	[5,7,20]
	95	Professionals in the care chain are informed/aware of each other’s expertise and tasks	[5,6,7,20]
	96	Education for professionals (continuous education)	[6,16,20,22,23]
	97	Training (joint or relating to collaboration)	[14,21,26]
	98	Inter-professional education	[6,7,9,20,23]
	99	Stimulating a learning culture and continuous improvement in the care chain	[7]

**Decision support**	100	Share registries and/or methods to track care/health	[5,14,23,26]
	101	Implementing care process-supporting clinical information systems	[7,26]
	102	Shared decision support	[26]
	103	Support/supervision for clinicians	[26]
	104	Clear communication strategies and protocols	[6]
	105	Standardised diagnostic and eligibility criteria	[5,7,14]
	106	Multidisciplinary and comprehensive assessment	[5,14]
	107	Developing criteria for assessing client’s urgency	[7]
	108	Case finding and use of risk stratification	[5]
	109	Common decision-support tools (practice guidelines, protocols)	[5,14,21,23]
	110	Multidisciplinary guidelines and protocols	[5,7,14]
	111	Existence of evidence-based clinical practice guidelines with automated tools to enforce their use	[6,7,20,29]
	112	Join planning	[5,7,9,14,20,21,26]
	113	Using a single client-monitoring record accessible for all care partners	[7]
	114	Using a protocol for the systematic follow-up of clients	[7]
	115	Information sharing, planned/organised meetings	[20]
	116	Shared decision-making and problem solving	[6]
	117	Shared-care protocols and evidence based practice guidelines	[5,6,9,14,18,20,23,29]
	118	Shared clinical records	[5,14,21]
	119	Integrated clinical pathways	[6]
	120	Decision aids to patients	[23]
	121	Providing understandable and client-centered information	[7]
	122	Assistance in accessing primary health care	[26]

**Clinical information system**	123	Intelligence systems for data collection	[18,22,23,25]
	124	Centralised system-wide computerised patient record system (data accessibility from anywhere in the system)	[6,20,23]
	125	Integrated electronic health records	[5,6,7,14,18,20,22,23,27]
	126	Electronic registry for planning care and risk-stratifying patients	[20]
	127	Technologies that support continuous and remote patient monitoring	[5,20,21]
	128	Reminders to clinicians and patients (e.g., medication management)	[5,23]

**Leadership**	129	Local leadership and long-term commitments	[5,6,7,27]
130	Leaders with a clear vision on integrated care	[27]
131	Distributed leadership	[5,6,7,21,25]
132	Managerial leadership	[5,6,7,9,18]
133	Visionary leadership	[9]
134	Clinical leadership	[5,9,25]
135	Organisational leadership for providing optimal chronic care	[20]
136	Conflict management	[9]
137	Reputation	[9]

**Governance**	138	Good governance	[9,18,22,23]
	139	Inter-organisational governance	[9]
	140	Inter-professional governance	[9]

**Performance & Quality**	141	Action oriented to understand and support more effective ways for improving quality and enabling change	[5,6,24,27,29]
	142	Collaborative learning in the care chain in order to innovate integrated care	[7]
	143	Involving leaders in improvement efforts in the care chain	[7]
	144	Involving client representatives by monitoring the performance of the care chain	[7]
	145	Using a systematic procedure for the evaluation of agreements, approaches and results	[5,7,9,20,23,25]
	146	Reaching agreements about the uniform use of performance indicators in the chain care	[7]
	147	Establishing quality targets for the performance of care partners	[7]
	148	Establishing quality targets for the performance of the whole care chain	[7]
	149	Installing improvement teams at care-chain level	[7]
	150	Evaluate outcomes	[24]
	151	Client satisfaction	[7,9,22]
	152	Performance management (common outcomes evaluation, performance indicator)	[5,7,9,18,21,22,24]
	153	Monitoring successes and results during the development of the integrated care chain	[7,20]
	154	Regular feedback of performance indicators	[9]
	155	Shared accountability/risk and responsibility for care	[5,6]
	156	Integrating incentives for rewarding the achievement of quality targets	[6,7,18,20,24]
	157	Gathering financial performance data for the care chain	[7]
	158	Gathering data on client logistics (e.g. volumes, waiting periods and throughput times) in the care chain	[7,21]
	159	Monitoring and analysing mistakes/near-mistakes in the care chain	[7]
	160	Monitoring whether the care delivered corresponds with evidence-based guidelines	[7]

**Organisational culture**	161	Shared vision and values for the purpose of integrated care	[5,6,9,18,25,27]
162	An integration culture institutionalised through policies and procedures	[5,6,7,9,20,27]
163	Organisational culture for providing optimal chronic care	[20,25]
164	Striving towards an open culture for discussing possible improvements for care partners	[7]
165	Linking cultures	[9]

**Contextual factors**	166	Population features (e.g., demographic composition)	[9,28]
	167	Advocacy	[16]
	168	Rurality of the area	[28]
	169	Environmental climate	[9]
	170	Environmental awareness	[9]
	171	Labour market	[9,28]

**Social capital**	172	Quality features of the informal collaboration	[9]
	173	Trust (on colleagues, caregivers and organisations)	[5,6,7,9]
	174	Reputation	[9]
	175	Interpersonal characteristics	[9]

From a quantitative point of view, about one third of the items (58) are supported by at least three contributions. Considering that some items are quite similar in the list, this is a conservative measure of the level of overlapping of the research findings and it can be interpreted as a degree of convergence on some important factors.

The categories with the highest concentration of elements are the Delivery system design (51), Community resources & policies (24), Decision support (23), Performance & quality (20), Healthcare system (17). While Governance (3), Social capital (4) Organizational culture (5), Contextual factors (6), and Clinical information system (6) show the lowest concentration.

More specifically, the category with the largest number of items concerns features of the service delivery design. This suggests that processes, logistics, and human resources management (e.g., multidisciplinary teamwork, staffing of professionals, training) have been widely investigated and represent a cornerstone of care integration. In addition, 43% (or 22) of the items classified in Delivery system design are identified by at least three different contributions. This is the highest level of convergence after Leadership (44%) and Clinical information system (67%); however, each of these last two categories groups a much lower number of items. One might conclude that research has already reported convincing evidence on some important aspects that should guide the design of service delivery to integrate care.

Focusing more on the contents, on the one hand several items grouped in the Delivery system design indicate the importance of centering service delivery on the needs of the patients (e.g. #53, 59, 60–63, 66). On the other hand, several items emphasize the need for standardizing (or foster uniformity of) specific aspects/tools that are paramount to ensure care quality and coordination across organizational boundaries and settings (e.g., #64, 65, 68, 72, 75, 77, 78), together with multi-/inter-professional collaboration (e.g., #71, 73, 74, 76, 79, 80).

At the system level two relevant policy areas are clearly identified: funding/financing mechanisms and priority setting coherent with the needs of the population (e.g., chronic conditions, older people) and the pillars of integrated care (e.g., cooperation between providers, synergic mobilization of community resources). The items grouped in the Contextual factors category reinforce importance of fine tuning interacting policies (e.g. health, environment, labor) with the actual needs and conditions of the population (e.g., demographic composition, orographic configuration of locations).

The categories Decision support and Community resources & policies group items that seem to point at setting the best conditions – by introducing changes and specific tools – to foster collaboration between professionals and organizations involved in the care delivery and help such actors to focus on patients’ needs and priorities.

The category Performance & Quality includes items that, rather than proposing specific technical solutions, indicate the need for fostering shared accountability on the results of the “care chain”. The few cases where end-points are proposed, they range from patient experience (e.g., satisfaction) to outcomes. This aspect, reinforced by the categories directly focused on soft aspects (i.e., Organizational culture and Social capital), suggests the importance of developing shared values to foster care integration.

## Conclusions

The literature evidence reviewed here has uncovered a range of elements and factors associated with successful care integration over the last decade. Moreover, the development of conceptual frameworks to understand and guide thinking on integrated care has grown and evolved over time.

However, the majority of contributions provide recommendations related to a smaller number of specific aspects that were found to be influential. Moreover, these were often derived in specific contexts/settings or with defined target patients, especially to those with chronic illnesses as opposed to those with comorbidities or wider health and social care needs. Few studies propose, and eventually validate, frameworks indicating key areas of intervention and/or analytical aspects to consider in order to foster care integration. They are mostly lists of key building blocks to integrated care, rather than frameworks supporting the process of implementation. In addition, the retrieved frameworks generally build on the findings of previous research, but each of them assumes either a diverse (though not necessarily alternative) perspective or different analytical degree. Interestingly, despite that our review focused on forms of integrated care addressing patients with chronic diseases and long-term conditions, we found that recent frameworks (more or less explicitly) assume a population health rather than a disease-based perspective. This dramatically increases the need for accurately defining client groups or populations, including people with non-medical conditions, profiling their needs and specificities, and manage the complexity resulting from this broad perspective.

When streamlining all the aspects identified in the available relevant literature, on the one hand, it is striking the high number of factors deserving attention; however, on the other hand, there is a clear area of convergence identified by those factors mentioned in several contributions. The two findings can be considered, respectively, evidence of dynamism and indicator of the degree of maturity the research field.

Researchers seem to have concentrated their attention on the service delivery design of integrated care, with a recent shift toward the notion of person-centeredness as a way of shaping any aspect of processes and interaction with patients and their caregivers. Nevertheless, contextual aspects seem to rank more and more high in the priorities of experts in the field. Perhaps, this is the result of the aforementioned shift toward a population health perspective that tends to increase the interdependencies between the different components of a health (and social) care system. It may also be that many experts view care integration as primarily a systemic or organizational activity rather than an approach (as defined) that co-ordinates care with and around people’s needs at the clinical and service level.

A limitation of our contribution is that we searched studies that propose frameworks or key factors for integrated care starting from three selected journals specialized in the field. This could have limited our capacity to identify all available studies related to the objective of this article. However, we privileged the pertinence of the scientific source and tried to compensate the aforementioned bias by snowballing the references of the included contributions and by involving some external scholars/experts to fill eventual gaps based on their extensive knowledge.

Our selectivity allowed us to perform an exhaustive, careful review of all the included material. The extracted key elements offer a useful basis for describing and/or reflecting on integrated care initiatives for chronic illnesses and long-term conditions set in different contexts. Nevertheless, it may be difficult to obtain accurate information on all of the important aspects so far identified. Therefore, it would be useful to develop a comprehensive framework that could synthetically describe care integration initiatives implemented in different contexts and allow for efficient comparisons highlighting relevant variabilities and context-dependencies.

## Footnotes

Formerly, International Journal of Care Pathways.
